# Supplementary figures and images for: Pairtools: From sequencing data to chromosome contacts
Source: PLoS Comput Biol. 2024 May 29;20(5):e1012164. doi: 10.1371/journal.pcbi.1012164 (PMC11164360; doi:10.1371/journal.pcbi.1012164)

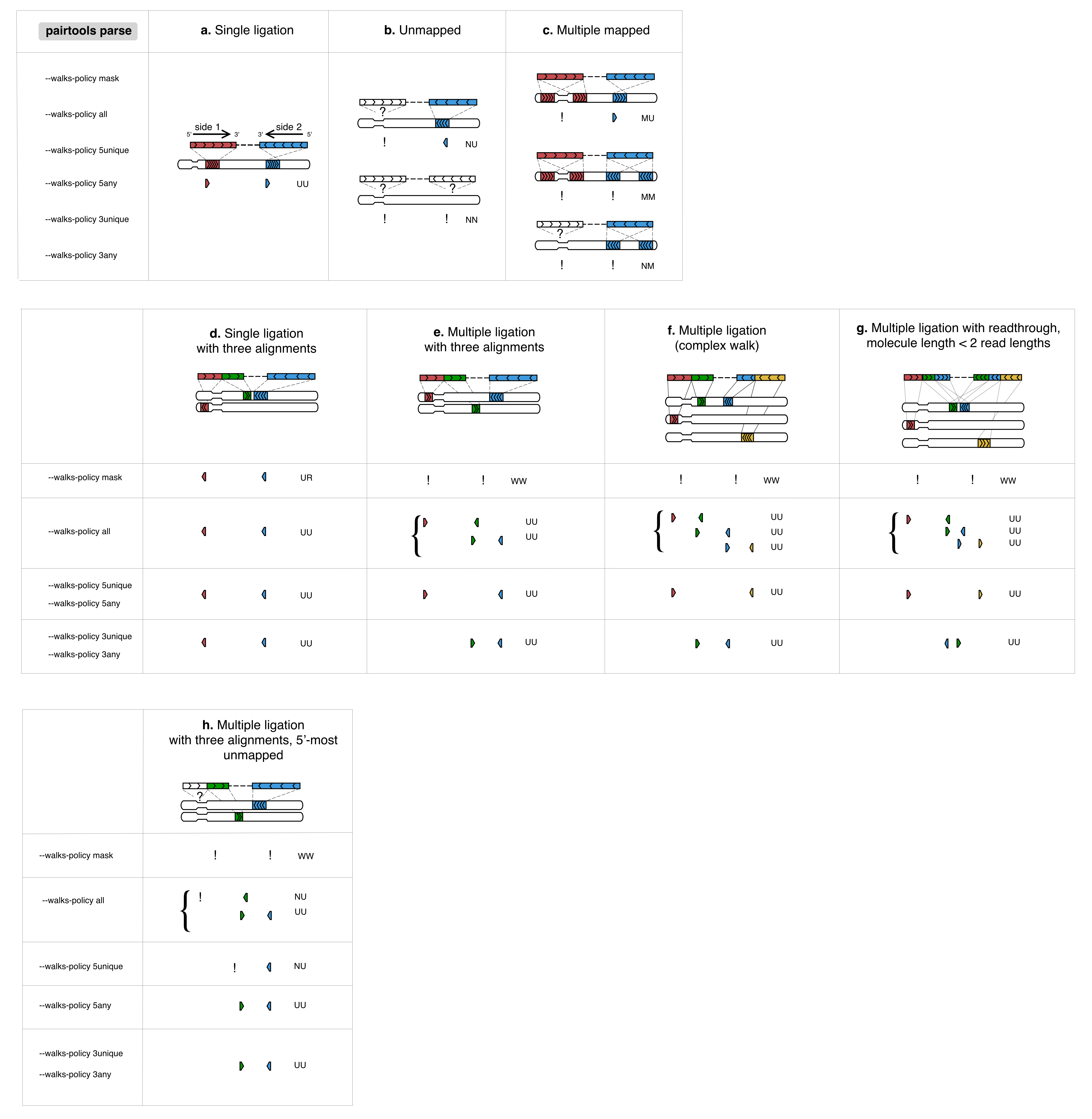

Supplement: S1 Fig — a-h. Different types of paired-end reads processed by parse. Notation is the same as in Fig 1b. a. Single contact with two alignments. Each side of the read contains a uniquely mapped alignment (red and blue). b. Unmapped pairs. Either one (top) or both (bottom) sides of the read do not contain segments aligned to the reference genome. c. Multiple mapped pairs. Either one (top, center) or both (bottom) sides of the read contain a segment that is mapped to multiple locations in the genome. d. Single contact with three alignments. One side of the read pair contains two segments that align to different genomic locations (red on the 5’ end and green on the 3’), while the second read side contains only one alignment (blue). If the green and blue alignments have opposite orientation, are located on the same chromosome and separated by the distance shorter than the typical molecule size, pairtools parse considers them part of the same DNA fragment. parse recognize “rescues” such pairs reports them as a contact between the red and the blue alignments. e. An “unrescuable” molecule with three alignments highlights the difference between walks policies. One side of the reads contains two unique alignments to distinct genomic locations (red and green). If the 3’ alignment (green) and the 2nd side alignment (blue) are too distant, do not have convergent orientations, or are in trans, the molecule cannot be “rescued” into a single contact and instead is considered as a two-contact walk.—walks-policy mask ignores such cases (W).—walks-policy all reports both ligations. `5unique`and `5any`report the two 5’-most alignments at each read side. `3unique`and `3any`report the 3’-most alignments at each read side. f. A molecule formed via three ligations. Both sides of the read contain two segments mapped to different unique locations.—walks-policy mask ignores such cases (W).—walks-policy all reports all three ligation events.—walks-policy 5unique and 5any report the two 5’-most alignmen [file pcbi.1012164.s001.tiff]

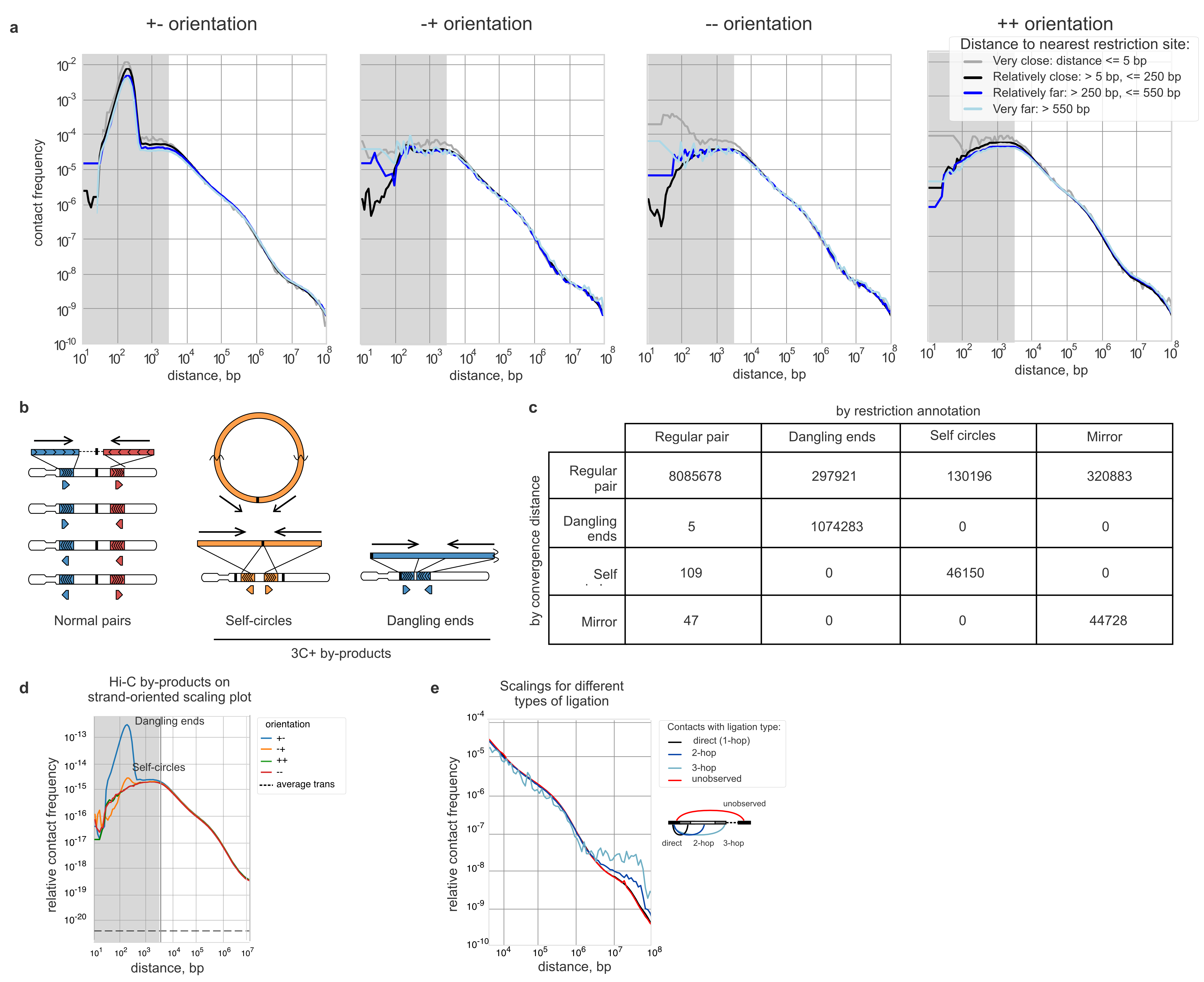

Supplement: S2 Fig — a. Orientation-dependent scalings for pairs grouped by distance to the nearest restriction site (DpnII Hi-C from [1]). Scalings are very close at genomic separations beyond the orientation convergence distance. b. Generation of normal pairs and by-products in 3C+ protocol. Normal pairs originate from distinct restriction fragments separated by at least one restriction site (in black). Pairs in self-circles and dangling ends are located on the same restriction site, either in divergent (self-circles) or convergent (dangling ends) orientation. c. Counts of pairs are categorized into four groups: regular pairs, dangling ends, self circles, and mirror pairs [43] for a test sample of 11 million pairs, by restriction enzyme annotation (columns) and convergence distance (rows). For restriction enzyme annotation, we considered dangling ends to be mapped to the same restriction fragment in the convergent orientation, self circles in the divergent orientation, and mirror pairs in the same orientation. For convergence distance annotation, we conservatively considered all the pairs below convergence distance as potential by-products and assigned them to each category by their orientation as for the restriction enzyme annotation. Both methods produce highly congruent filtration, as seen by the relatively smaller number of off-diagonal pairs. d. Scaling with prominent peak of self-circles and dangling ends. A short-range peak in pairs mapped to opposing strands facing away from each other (divergent) is a sign of self-circled DNA, while a short-range peak in pairs mapped to opposing strands facing each other (convergent) pairs is a sign of dangling ends. e. Scalings for direct, indirect (2- and 3-hops), and unobserved contacts. Note that multi-hop contacts have a flatter scaling, potentially indicating more ligations in the solution [22,23]. (TIFF) [file pcbi.1012164.s002.tiff]
