# Supplementary material for: Pairtools: From sequencing data to chromosome contacts
Source: PLoS Comput Biol. 2024 May 29;20(5):e1012164. doi: 10.1371/journal.pcbi.1012164 (PMC11164360; doi:10.1371/journal.pcbi.1012164)
Supplement: Attachment — Submitted filename: 240328-pairtools-PLoS-response-to-reviewers.pdf [file pcbi.1012164.s003.pdf]

Dear Editors and Reviewers,

Thank you for your thorough review and insightful comments on our manuscript. We have made substantial revisions to address all of your suggestions, including:

(1) adding a discussion on the homolog bias in pairtools phase;

(2) clarifying the role of filtering in Hi-C data processing and enabling automated filtering of non-informative Hi-C byproducts;

(3) simplifying the manuscript by focusing on pairtools parse and removing the sections on parse2 to be explored in future work;

(4) adding a new documentation page on common Hi-C analysis workflows and clarifying the key settings of pairtools;

(5) updating pairtools to use a clearer notation for reporting walk-related information and clarifying the role of the --walks-policy parameter.

The point-by-point responses are provided below. We believe these changes have significantly improved the manuscript and hope that it now meets the standards for publication in your journal.

Sincerely,

Open2C.

Below, you can find the original notes by reviewers are in black fond and our response is in blue.

Reviewer #1: The Open2C consortium presents a manuscript in which they describe a computational tool (pairtools) that parses sequencing data from a Hi-C experiment that has been mapped using a standard pipeline (e.g. bwa or minimap). It parses the raw mapping file that can be used as input for cooler, which can generate Hi-C contact matrices that are used for further analysis and viewing of the Hi-C data. In that sense it serves a function similar to SAMtools. The paper is clear summary of what the software can do. There is also a nice comparison to similar tools, including memory and speed benchmarking.

I have no major comments for the paper. I have a few suggestions for improvement.

1.1 - Can the authors discuss whether pairtools phase has a bias for the reference allele. This can be an issue with allele-specific reads and specific tools have been developed to counteract this (e.g. WASP, PMID: 26366987). I can imagine that for short fragments this can be an issue.

Thank you for this suggestion! Indeed, homolog bias is a crucial aspect of any homolog-resolved method. We revisited the homolog-resolved studies performed with pairtools (Erceg et.al. 2019, AlHaj Abed et.at. 2019) and found the homolog bias to be between 1% and 3%. We attribute these low biases to two factors: (1) our approach to calling homologs, where reads are aligned to “concatenated” genomes containing both homologs, treats them equally, and avoids introducing biases at the mapping stage; (2) in this particular study, the SNVs for paternal and maternal homologs had equal quality, as we re-sequenced both parental strains and the F1 progeny. Conversely, in situations where the two sets of SNVs have different qualities and/or come from different sources, one should expect higher homolog biases. We have added the following paragraph to the manuscript:

“

The approach of pairtools to resolving homologs is designed to minimize homolog biases: when reads are aligned to both homologs simultaneously, both homologs are treated equally. For example, in the two studies that introduced the approach behind pairtools phase 10,52, the observed homolog bias was around 1-3%. Similarly important for this low bias was the fact that this study re-sequenced both parental strains as well as the genome of their F1 progeny, thus obtaining high-quality SNVs for both homologs. Otherwise, potential differences in the SNV quality between the two homologs (for example, if two parental strains were sequenced with different quality) could introduce homolog bias.

”

1.2 - The authors discuss multi-contact 3C+ methods, they should also include Tri-C (Hughes lab), MC-4C (de Laat lab) and Nano-C (Noordermeer lab)

1.3 - Hi-C data is often binned, but also computational methods exist that do not use binning: binless (PMID: 31028255) and shaman (https://www.biorxiv.org/content/10.1101/187203v1)

(Addressing 1.2 and 1.3): We have updated the manuscript to mention these citations.

Reviewer #2: Abdennur and collegues present here a new python package, named pairtools, to efficiently process Hi-C based sequencing data from raw sequencing reads, to normalized contact matrices (in .cool format). Compared to other existing solutions, pairtools offers the possibility to detect multiple proximity ligation envents (called walk) which are particularly interested for long-reads sequencing technologies or to explore more complex ligation events. The python package provides a CLI which make the different steps easy to implement in a bioinformatics pipeline. The code is well packaged, easy to install and well documented. For these reasons, I think pairtools could become a valuable standard in the field of Hi-C processing in the coming years.

Overall the manuscript is well written but can be a bit difficult to follow for non experts (especially the part on the 'walk' reads). Here are a few comments on the manuscript and the tools itself ;

2.1 - The minimal pairtools-based pipeline is presented in the manuscript and is defined as mapping | parse | sort | dedup.
I think this view is misleading because it does not include any filtering step(s) to remove non valid 3C products.
The end-users could think that 90% of the sequenced reads are good 3C products while this is usully not the case.
I think the 'pairtools select' step should be part of the standard pipeline to filter non-valid 3C products (regardless how they are filtered).

We agree that filtering is essential to Hi-C processing pipelines and that the manuscript needs to discuss this issue in more detail. However, we omit filters from **the minimal pipeline** and defer discussion of filtering until later in the manuscript for two reasons:

1) The philosophy of the Open2C tool suite is to avoid early filtering, instead generating and storing the most complete (i.e., unfiltered but tagged) versions of datasets. This approach helps prevent data loss and maintains a comprehensive overview of the dataset. For example, identifying the strand convergence distance for filtering Hi-C by-products necessitates access to unfiltered data.
2) The minimal pipeline does, in fact, filter data, albeit downstream of pairtools. Specifically, cooler and cooltools automatically filter non-informative Hi-C by-products by ignoring the first two diagonals of the matrix in all computations. These default settings are typically sufficient for 4-bp cutter Hi-C and Micro-C at resolutions >= 1kb.

The revised manuscript now discusses filtering in the chapter "Stats and scaling" after introducing all the concepts and tools necessary for understanding filtering:

“

*The orientation convergence distance reported by **pairtools stats** can also be used to remove all Hi-C byproducts from binned output conservatively:*

```
pairtools stats library.nodups.pairs.gz -o library.stats
CONV_DIST=`grep "summary/dist_freq_convergence/convergence_dist" library.stats | cut -f2`
pairtools select "(chrom1!=chrom2) or (abs(pos1-pos2)>=${CONV_DIST})" library.nodups.pairs.gz \
        | cooler cload pairs -c1 2 -p1 3 -c2 4 -p2 5 chromsizes.txt:1000 - output.1000.cool
```

Such filtering is, however, typically unnecessary as cooler and cooltools by default ignore the first two diagonals in most computations, which is sufficient to remove by-products of 4bp-cutter Hi-C and Micro-C at resolutions >=1kb.

"

2.2 - The bwa mem options are important for Hi-C data processing. I would suggest to specify which options to use in the command line exemple L102.

We agree. The updated version of the manuscript now mentions aligner flags:

"Importantly, aligners must align the two reads of a pair independently (i.e., avoid 'pair rescue'). In the case of bwa mem, adding the -SP flags ensures this behavior."

We have also updated the online documentation to mention aligner settings.

2.3 - After reading the manuscript, I cannot see in which cases the 'parse' command should be used instead of the 'parse2' command. It seems that 'parse2' could completly replace both commands ? if so, I would simplify the message by only presenting the 'parse2' command.

2.4 - In supFig1h, we can see that the behavior of the --report-orientation option in parse2 is different than in the parse command. What is the reason for that ? Simple recommandation or use case about which options to use would help the user.

2.5 - To better understand the interest (and the differences) of parse versus parse2, would it makes sense to always illustrate how the command will handle a single ligation and a multiple ligation. For instance, in Fig1a, I guess in a case of multiple ligation, the parse command will return a contact between the red and yellow part, while is it not reported with the parse2 command, is that correct ?

2.6 - The walk parsing strategy is not clearly explained in the Figure 2 legend. What is the message here ?

(Addressing questions 2.3-2.6) We agree with the reviewers that the distinction between 'parse' and 'parse2' complicated the original version of the manuscript. Upon further reflection, we have realized that a thorough treatment of Hi-C walks and optimizing software for analyses of such walks extends beyond the current scope of this manuscript. The current version of parse2 represents the first step in this direction, which may still undergo major changes in the future. As

such, we have removed 'parse2' and its related sections to provide a more focused study. We will explore this functionality in greater detail in future work.

2.7 - Could you illustrate the interest of 'paritools header' with a concrete use case ? In which cases this function could be useful ?

Thank you - indeed, the use case for pairtools header may not be clear for our readership. We have added the following short description to the corresponding section of the manuscript:

Pairtools header helps to fix .pairs files that were generated by scripts or software that do not fully comply with the .pairs format specifications and have missing or improperly formatted headers.

2.8 - I always had in mind that PCR duplicates are reads that start/end exactly at the same genomic positions (and align in the same way on the genome). To my knowledge, this is the definition used by picard or samtools to remove duplicates. I'm not sure to understand, why for Hi-C data it could be interesting to account for potential losses of a few nucleotides ? what is the technical rational behind this ?

Thank you for raising this issue. During the preparation of the answer to this question, we carefully examined several publicly available datasets and visualized the distributions of distances between molecules with similar alignments on both sides. Interestingly, we found no evidence of even minor (+/- 1bp) variability in the 5'-positions of PCR duplicates. Based on this result, we have updated the default behavior of pairtools dedup to require only perfect matches between molecules to call them PCR duplicates.

Additionally, we have revised the text of the paper to reflect this change. We no longer suggest that PCR duplicates in standard Hi-C protocols may have positional mismatches. Instead, we emphasize an alternative use case for allowing mismatches in dedup: duplicate detection in single-cell protocols that involve whole genome amplification. The updated version of the sentence now reads as follows:

"*To accommodate non-standard protocols, e.g. some varieties of single-cell Hi-C [15,16], where the amplification step occurs between ligation and sonication,* ***pairtools dedup*** *can detect duplicated pairs even when their mapped positions do not match exactly.* "

2.9 - The command 'pairtools scaling' is interesting to validate the QCs of an experiment and to define which minimal distance between two interactors could be used to remove those artefacts. However, this is not really compatible with a standard bioinformatics worflow to automatically process Hi-C data. Here again, recommandations or exemple of the 'select' command could be useful for the users.

2.10 - L297-298. The authors mentionned the interest for digestion Hi-C protocols to have access to the fraction of unwanted 3C products like self-circle, dangling-end, and mirror reads.

Does pairtools provide those statistics ? or is there a way to easily get the information?

(Addressing questions 2.9-2.10) Thank you for this useful suggestion! We have updated *pairtools stats* to calculate and report the orientation convergence distance. As we show in the answer to point 2.1., this distance can be extracted via Unix command line tools and used in automated data processing pipelines. Following the suggestion from point 2.10, we also report the number of cis reads with separations below the convergence distance, split by four possible read orientations. We additionally express these numbers as a fraction of (a) all, (b) uniquely mapped and deduplicated, and (c) cis pairs. Together, these numbers give an estimate of the fraction of non-informative Hi-C by-products, such as self-circles and dangling ends.

2.11 - The comparison of the performance of the different tools is interesting but, to me, not the most important information for the end-users. Did the authors compare the performance of the tools in terms of valid ligation products detected ? is there any big differences ? This could be a way to illustrate the interest of the 'walk' reads strategy which is one of the interest of pairtools compared to the other tools. If not major difference is observed on the final list of valid ligation products between the different tools, I think this should be also mentioned.

We agree that performance is just one of many factors considered by users when choosing tools. Comparing the ability of different tools to detect valid ligation products is indeed important, but a thorough and objective comparison would require significant time and effort, and lies outside of the scope of this manuscript. More importantly, a quick comparison by us alone would likely be biased; to ensure fairness, the creators of the other tools should be involved, as they have the deepest understanding of their software and can help select optimal parameters. Thus, a detailed comparison of the tools' capabilities is better suited for a dedicated benchmarking study by an independent 3rd party or a competition, similar to those in the field of protein folding (e.g. CASP or CAPRI).

I've tested pairtools on some MicroC data and did not had any major issue in installing it through conda and even using it in practice. I was able to get some first results in a few hours. Here are a few questions I had while running the CLI ;

2.12 - The package itself is very flexible, even maybe too flexible for beginners. I would appreciate to have some clear guidelines about what would be a typical or standard worflows for the most common Hi-C protocols, and which parameters to use for each CLI step.

Indeed, the extensive feature set of pairtools and its flexibility can be overwhelming for new users. To make it easier to get started, we have added a new page to the online documentation that accomplishes the following:

1. Provides a step-by-step guide for setting up the minimal Hi-C pipeline, utilizing the Open2C toolset.
2. Explains the main parameters of pairtools and the factors to consider when choosing the optimal values.

The new section of documentation can be found here: https://pairtools.readthedocs.io/en/latest/protocols_pipelines.html . We will continue to update it regularly based on questions received on the Pairtools issue tracker and Slack channel.

2.13 - Running 'pairtools stats', I saw in the statistics file some pair types I was not able to defined ;
pair_types/UU 147151119
pair_types/Uu 43246049
pair_types/uU 43414173
pair_types/uu 14042597
pair_types/RU 1341742
pair_types/UR 1346450
What is the difference between u and U ? is there any documentation on that ?
I also noticed that in the pairs file, I only have UU ... and no uu ?

We thank the reviewer for pointing out the confusion created by the difference between lower- and upper-case pair types. At the time of submission, *pairtools parse* used lowercase in pair types to indicate read sides containing multiple alignments (i.e. sides that could give rise to walks as thus requiring walk-policy for their interpretation). This suggestion highlights that (a) this notation was too confusing and difficult to interpret and (b), more importantly, could interfere with pair type-based filtering. To address this, we updated *pairtools parse* to use only upper case letters in pair types. We moved the walk-related information into three optionally reported columns: (i) “read_side”, i.e. which side of a paired-end read the alignment originates from, (ii) “algn_idx”, the 0-based index of the alignment in the read side, counting from the 5’-end, (iii) “same_side_algn_count”, i.e. the total number of alignments in the same read side. With this information, users will be able to filter out pairs originating from walks.

2.14 - The 'walks-policy' option of the parse command is not easy to understand. By default, I would used the '--walks-policy 5unique' for standard Hi-C protocol (digestion Hi-C) but I'm not sure to see how this option will impact the final results ?

Thank you for spotting this omission. We have updated Supplementary Figure 1 to include panels describing the effects of different walk policies in a few typical scenarios.

Have the authors made all data and (if applicable) computational code underlying the findings in their manuscript fully available?
The PLOS Data policy requires authors to make all data and code underlying the findings described in their manuscript fully available without restriction, with rare exception (please refer to the Data Availability Statement in the manuscript PDF file). The data and code should be provided as part of the manuscript or its supporting information, or deposited to a public repository. For example, in addition to summary statistics, the data points behind means, medians and variance measures should be available. If there are restrictions on publicly sharing data or code —e.g. participant privacy or use of data from a third party—those must be specified.

Reviewer #1: Yes

Reviewer #2: Yes

PLOS authors have the option to publish the peer review history of their article (what does this mean?). If published, this will include your full peer review and any attached files.

If you choose “no”, your identity will remain anonymous but your review may still be made public.

Do you want your identity to be public for this peer review? For information about this choice, including consent withdrawal, please see our Privacy Policy.

Reviewer #1: No

Reviewer #2: No
